# Brain metastasis screening in the molecular age

**DOI:** 10.1093/noajnl/vdad080

**Published:** 2023-07-12

**Authors:** Joanna K Tabor, Amanda Onoichenco, Vinayak Narayan, A Gabriella Wernicke, Randy S D’Amico, Morana Vojnic

**Affiliations:** SUNY Downstate College of Medicine, Brooklyn, NY, USA; Department of Neurological Surgery, Lenox Hill Hospital, Donald and Barbara Zucker School of Medicine at Hofstra/Northwell, New York, NY, USA; SUNY Downstate College of Medicine, Brooklyn, NY, USA; Department of Neurological Surgery, Lenox Hill Hospital, Donald and Barbara Zucker School of Medicine at Hofstra/Northwell, New York, NY, USA; Department of Neurological Surgery, Lenox Hill Hospital, Donald and Barbara Zucker School of Medicine at Hofstra/Northwell, New York, NY, USA; Department of Radiation Medicine, Lenox Hill Hospital, Donald and Barbara Zucker School of Medicine at Hofstra/Northwell, New York, NY, USA; Department of Neurological Surgery, Lenox Hill Hospital, Donald and Barbara Zucker School of Medicine at Hofstra/Northwell, New York, NY, USA; Northwell Health Cancer Institute at Lenox Hill Hospital, Donald and Barbara Zucker School of Medicine at Hofstra/Northwell, New York, NY, USA

**Keywords:** brain metastasis, driver mutations, molecular characterization, screening, surveillance

## Abstract

The incidence of brain metastases (BM) amongst cancer patients has been increasing due to improvements in therapeutic options and an increase in overall survival. Molecular characterization of tumors has provided insights into the biology and oncogenic drivers of BM and molecular subtype-based screening. Though there are currently some screening and surveillance guidelines for BM, they remain limited. In this comprehensive review, we review and present epidemiological data on BM, their molecular characterization, and current screening guidelines. The molecular subtypes with the highest BM incidence are *epithelial growth factor receptor*-mutated non-small cell lung cancer (NSCLC), *BRCA1*, triple-negative (TN), and HER2+ breast cancers, and *BRAF*-mutated melanoma. Furthermore, BMs are more likely to present asymptomatically at diagnosis in oncogene-addicted NSCLC and *BRAF*-mutated melanoma. European screening standards recommend more frequent screening for oncogene-addicted NSCLC patients, and clinical trials are investigating screening for BM in hormone receptor+, HER2+, and TN breast cancers. However, more work is needed to determine optimal screening guidelines for other primary cancer molecular subtypes. With the advent of personalized medicine, molecular characterization of tumors has revolutionized the landscape of cancer treatment and prognostication. Incorporating molecular characterization into BM screening guidelines may allow physicians to better identify patients at high risk for BM development and improve patient outcomes.

Brain metastases (BM) are the most common intracranial tumors in adults and a major cause of morbidity and mortality, affecting an estimated 9%–17% of all cancer patients.^1–3^ The most commonly associated primary cancers are lung cancer, breast cancer, and melanoma, accounting for 67%–80% of BM cases.^2,4,5^ Despite an increasing incidence of BM due to improved cancer survival rates, traditional imaging techniques have limitations, and there is no routine screening in place.^2^ This may affect patient outcomes, as BM can significantly impact patient quality of life and survival, making early detection and treatment crucial.

Recent advances in our understanding of the molecular basis of BM have led to the development of more personalized and effective therapeutic interventions.^6^ With the advent of personalized medicine, molecular characterization of tumors has revolutionized the landscape of cancer treatment, providing insight into the biology, oncogenic drivers, and targeted therapies of these tumors as well as their prognosis relating to BM development.^7^ In light of these developments, the need for updated screening guidelines has become increasingly clear.^8–11^

Current BM screening guidelines are based on National Comprehensive Cancer Network (NCCN) recommendations. Screening for BM is recommended for some stages of non-small cell lung cancer (NSCLC), and metastatic or advanced-stage melanoma, while small cell lung cancer (SCLC) requires screening at diagnosis and throughout the disease course.^8,10,11^ Screening brain imaging for BM is recommended for breast cancer patients only if they develop neurologic symptoms.^9^

Recent studies have reevaluated the indications for BM screening, and there is evidence to support the use of molecular testing results in informing these practices.^12^

Our increasing understanding of the molecular basis of BM and advances in primary tumor treatment have provided new opportunities to improve patient outcomes. As targeted therapies further improve patient outcomes and prolong survival, the increasing incidence of BM among these patients has become a growing concern, and studies exploring BM prevention and early detection have become increasingly important. Therefore, in this timely and comprehensive review, we present epidemiological data on BM and address the role of molecular information in guiding screening practices. We aim to highlight the importance of incorporating tumor sequencing into BM screening guidelines, to better identify patients at high risk and improve patient outcomes.

## Epidemiology of Brain Metastases

BM are a growing concern for patients with cancer, affecting up to 14.3 per 100 000 people and up to 1 in 5 cancer patients.^13–15^ Up to 80% of BM may be attributed to lung cancer, breast cancer, and melanoma. Lung cancer remains the leading primary source of BM comprising 39%–56% of all cases and is driven by the high prevalence of primary lung cancer. Similarly, the high incidence of primary breast cancer makes it the second leading cause of BM accounting for 13%–30% of cases despite it being relatively less likely to go to the brain. While melanoma is understood to be the most likely to metastasize to the brain, it only accounts for 6%–13.8% of BM.^2^

The incidence of BM varies based on the age at diagnosis, with the highest incidence among patients diagnosed with primary breast cancer between the ages of 20–39, primary lung cancer between the ages of 40–49, and primary melanoma between the ages of 50–59.^4^ A portion of patients present with BM at their initial cancer diagnosis and rates of BM at initial diagnosis have increased over time likely secondary to current screening guidelines. In an assessment of the SEER database from 2010 to 2013, rates of BM at initial diagnosis were highest for melanoma (28%), followed by lung cancer (16%–27%), and breast cancer (8%).^16^

However, the incidence of BM is on the rise due to factors such as improved cancer treatment, increased overall survival, and greater awareness of BM.^13,14^ The aging population is also a factor, as BM are more common in elderly patients.^17,18^ However, there is still a lack of comprehensive data on BM. This is partly because existing cancer databases prioritize primary cancers, and a proportion of asymptomatic BM cases are only diagnosed postmortem.^19–21^ Over the past decade, the BM median overall survival (OS) rates have increased to 7 months, but still vary greatly depending on primary tumor characteristics.^22^ (Table 1, Table 2)

**Table 1.** Epidemiology of brain metastases (BM)

Primary Tumor Site	Percentage of all BM (%)	Incidence BM at Primary Cancer Diagnosis (%)	Lifetime risk of BM (%)	Time to Development of Brain Metastasis (months)	Median Overall Survival After BM (months)
Lung	39–56 (Nayak et al.^2^) 43.9 (Kavouridis et al.^26^)	15.5–26.8 (Cagney et al.^16^)			
NSCLC	22–44 (Nayak et al.^2^)			22.6 (Wang et al.^25^)	
SCLC	6–18 (Nayak et al.^2^)	10–20 (Nayak et al.^2^)	50–80 (Nayak et al.^2^)	9–11 (Steindl et al.^22^)	5–9 (Steindl et al.^22^)
Breast	13–30 (Nayak et al.^2^) 14.4 (Kavouridis et al.^26^)	7.58 Cagney et al.^16^) 7.2 (Darlix et al.^50^)		29.7 (Simsek et al.^51^) 39 (Sperduto et al.^52^) 46 (Shen et al.^53^)	5.5 (Tham et al.^54^) 7.2 (Simsek et al.^51^) 14.1 (Shen et al.^53^) 16 (Sperduto et al.^52^)
Melanoma	6–10 (Nayak et al.^2^) 13.8 (Kavouridis et al.^26^)	28.2 (Cagney et al.^16^)	40–50 (Chukwueke et al.^66^) 44 (Davies et al.^67^)	32 (Sperduto et al.^83^) 37.2 (Fife et al.^71^) 30.5 (Qian et al.^69^)	10 (Sperduto et al.^83^) 13 in 2015–2019 cohort 7 in 2010–2014 cohort (Bander et al. 2021)^72^ 4.83 in 2011–2016 cohort (Frinton et al.^80^) 4.1 months in 1985–2000 cohort (Fife et al.^71^)

**Table 2.** Incidence and Prognosis of brain metastases (BM) According to Molecular Characteristics

Primary Tumor Site	Molecular Subtype or Mutation	Incidence BM at Primary Cancer Diagnosis	Lifetime Risk of BM	Time to Development of Brain Metastasis (months)	Median Overall Survival After BM (months)
NSCLC	EGFR	70 (Kelly et al.^29^)			30 (Tomasini et al.^28^) (With surgery and/or SRS)
	ALK	28 (Griesinger et al.^27^)			49.5 (Griesinger et al.^27^)
	KRAS	55.3 (Tomasini et al.^28^)			22 (Tomasini et al.^28^) (With surgery and/or SRS)
Breast	HER2+	11.37 (Martin et al.^58^)	40–50 (Ramakrishna et al.^57^) 50 (Sperduto et al.^52^)	39 (Steindl et al.^22^) 20.4 (Simsek et al.^51^) 46 (Shen et al.^53^)	5.3 (Simsek et al.^51^) 18 (Shen et al.^53^) 13.1–16.5 (Syriac et al.^64^) 13.1 (Darlix et al.^50^)
	HR+			75 (Steindl et al.^22^) 39.8 (Simsek et al.^51^)	7.6 (Simsek et al.^51^) 7.1 (Darlix et al.^50^)
	Triple Negative	11.45 (Martin et al.^58^)	25–46 (Sperduto et al.^52^)	30 (Steindl et al.^22^) 22.8 (Simsek et al.^51^) 27 (Shen et al.^53^)	3.5 (Simsek et al.^51^) 10 (Shen et al.^53^) 4.4–4.9 (Syriac et al.^64^) 4.4 (Darlix et al.^50^)
	BRCA1		44.7 (Garber et al.^55^)		
	BRCA2		16.7 (Garber et al.^55^)		
Melanoma	BRAF				3.7 (Frinton et al.^80^) 9 (Sperduto et al.^83^)

## Epidemiology, Molecular Characterization, and Screening Guidelines for Common Brain Metastases

### Lung Cancer

Lung cancer is the most common primary source of BM, accounting for up to 56% of all cases. NSCLC is the most common subtype, accounting for 24%–44% of BM, with adenocarcinomas contributing to 50% of NSCLC BM. SCLC accounts for only 6%–18% of BM but is more aggressive, with an estimated 50%–80% of patients developing BM, and 10%–20% of SCLC having BM at diagnosis.^2,20^ While the incidence of SCLC and subsequent BM has been decreasing, patients with BM continue to show worse survival compared to those without BM.^23^ The median interval from primary diagnosis to BM in NSCLC is estimated to be 22.6 months, while in SCLC it is only 9–11 months.^22,24,25^ The OS following the detection of BM in SCLC patients ranged from 5 to 9 months, depending on the presence of neurological symptoms.^22^

Driver mutations contribute to the growth of cancer cells and some have been linked to better survival in NSCLC BM patients.^26^ Up to 69% of NSCLC BM patients present with driver gene mutations including Epithelial growth factor receptor (*EGFR*), tumor protein 53 (*TP53*), Kirsten Rat Sarcoma viral oncogene homolog (*KRAS*), phosphatidylinositol-4.5-bisphosphate 3-kinase catalytic subunit alpha (*PIK3CA*), anaplastic lymphoma kinase (*ALK*), and rearranged during transfusion (*RET*).^25^ The gene mutations associated with the highest incidence of BM in NSCLC are mutations in *EGFR* (70%), *KRAS* (55%), and ALK(28%).^27–29^ While representing only a minority of NSCLC, *MET* Exon 14 skipping mutations (3%–4% of NSCLC) have been associated with a 17% incidence of BM at diagnosis and a lifetime prevalence of 36%.^30^*RET* fusions are also rare and found in 1%–2% of NSCLC, with 25% of patients having BM at diagnosis and 46% developing BM throughout their disease course.^31^ Lastly, *ALK* fusion-positive NSCLC (3%–7%) patients have a high risk of BM development with a reported incidence of 28% at diagnosis and 58% at 3 years.^27^

Patients with *EGFR*-mutant NSCLC are more likely to develop BM (70%) than patients with a WT (wild type) *EGFR* (38%).^29^ Although, *EGFR*-mutant NSCLC had longer OS than *EGFR* WT, likely secondary to the availability and efficacy of *EGFR*-targeted therapy, the increased incidence of BM is not entirely explained by this difference in survival.^32^ Notably, studies show that *EGFR-*mutant NSCLC has a higher incidence of BM at the time of diagnosis, suggesting an inherent propensity for BM in these cancers compared to WT.^33^

*KRAS* mutations are associated with smoking history, but can occur in both smokers and nonsmokers and are associated with an aggressive phenotype and occur at a higher proportion in BM groups (43%, of which 63% is G12C mutation) than in non-BM groups (32.3%).^34,35^

While the status of *EGFR* and *KRAS* appears to have no effect on the time from primary diagnosis to the development of BM, other gene mutations in NSCLC have been associated with significantly shorter time to development of BM, including those activating PI3K signaling.^25^

The PI3K pathway mediates cell motility, invasion, and metastasis, and NSCLC patients with activated PI3K/mTOR/AKT pathway (mutations in *PIK3CA*, *PTEN*, and *STK11*) in their primary tumors have shown shorter BM-free survival.^25^

Interestingly, the distribution of mutations varies by geography and ethnicity, with higher *EGFR*-mutation rates in Asian patients and higher *KRAS* mutation rates in Caucasians. In a study of NSCLC tumors in Chinese patients with BM, *EGFR* was predominantly mutated (51%), followed by *TP53* (41%) and *CDKN2A/CDKN2B* (30%), while *KRAS* mutations appeared least frequently (18%).^25^ Other studies including those of a Japanese population, suggest *EGFR*-mutation rates as high as 63% in NSCLC primary tumors and corresponding BM.^36^ Conversely, studies of NSCLC and adenocarcinoma in primarily Caucasian patients with presumed European ancestry showed a lower prevalence than Asian patients for *EGFR*-mutation (3.9%–11.27%), comparable for *TP53* (46.1%) and *CDNKA* (22.4%), and higher for *KRAS* mutation (33.1%–38.2%).^28,37^ In Caucasians, *KRAS* mutation appears to be more common than *EGFR* mutation, but BM incidence is higher in *EGFR*-mutation groups.^28^

In general, despite having a greater association with BM (up to 38%), *EGFR* mutations have a favorable prognosis compared with *KRAS* mutations with *EGFR*-mutant patients having a longer median OS than patients without the mutation and a longer OS than untreated *KRAS*-mutant patients.^28,38^ However, *KRAS*-mutant BMs are less likely to recur after local treatment than *EGFR*-mutant or *KRAS/EGFR* WT tumors.^28^ While *KRAS* and *EGFR* mutations rarely coexist in lung adenocarcinomas, *KRAS* mutations interfere with the efficacy of EGFR-tyrosine kinase inhibitors, and acquired *KRAS* mutations are known to induce resistance to EGFR inhibitors.^39^

Importantly, current molecular characterization focuses on NSCLC and studies on molecular predictors of BM in SCLC primary tumors are sparse, due to the lack of targetable oncogenic drivers and lack of response to targeted therapies. In addition, there is no appropriate BM prevention method in NSCLC, while BM in SCLC is historically thought to be preventable by prophylactic cranial irradiation (PCI) in spite of its negative neurocognitive effects.^40^

Current screening guidelines vary depending on the type of lung cancer. For limited-stage SCLC, PCI was historically recommended after a complete or partial response to primary treatment given its high incidence of metastasizing to the brain.^41^ However, this practice has gone out of favor due to newer imaging methods and advances in radiation therapy. Despite this, current NCCN guidelines continue to recommend PCI after a complete or partial response to primary treatment in limited-stage SCLC, and in some cases of extensive-stage SCLC.^11^ Importantly, these recommendations were made before current staging approaches with MRI and PET scanning were available, and evidence has demonstrated no benefit in OS after PCI for SCLC.^42^

Current screening for SCLC BM and brain MRI or CT with contrast are recommended after primary treatment, followed by surveillance MRI every 3–4 months for 1 year, then every 6 months for 2 years, and then as indicated by clinical status regardless of PCI status.^11^

For NSCLC, screening is currently recommended at initial staging for stages II and above, but not for stage IA (T1N0) and is optional for stage IB (T2aN0).^8^ However, patients with stage IA NSCLC are still at risk for BM and up to 3% of neurologically asymptomatic patients will present with BM at the initial diagnosis.^43^ Lymph node involvement is predictive of BM, and patients without nodal involvement are less likely to develop BMs compared to those with N1 or N2 disease.^44^ That said, studies of stage I NSCLC undergoing brain imaging with CT and/or MRI prior to lung disease resection, found that the incidence of asymptomatic BM amongst these patients (1.4%–2.4%) was too low to justify routine screening.^44,45^ As a result, preoperative brain imaging for stage II NSCLC patients undergoing surgical resection of their disease is recommended as these patients can have concurrent asymptomatic BM.^46^

Patients with adenocarcinoma or large cell carcinoma have a higher risk of developing BM compared to those with squamous cell carcinoma and recent evidence suggests that *RB1* and *ALK* alterations may also predict BM suggesting more frequent MRIs in these patient populations.^46,47^

There are currently no guidelines for follow-up surveillance imaging for patients who initially screened negative for BM, but up to 50% of these patients may develop intracranial spread later in their disease course.^48^ Stage III–IV and *EGFR*-mutant adenocarcinomas are risk factors for BM development and may warrant surveillance imaging a year after the initial evaluation.^49^ Currently imaging after initial screening is only recommended after the development of neurologic symptoms.

### Breast Cancer

Breast cancer is the second leading cause of BM, responsible for 13%–30% of cases.^2,26,50^ The interval between primary diagnosis and the development of BM can vary widely, with some sources reporting a median time from primary diagnosis to development of BM of 29.7–46 months.^51–53^ However, this can depend on the age of diagnosis, with younger patients showing a shorter time to BM compared with older patients, occurring at a median of 28 and 51 months in those under age 46 and between ages 64–92, respectively.^52^ Intervals between primary diagnosis and BM can also vary depending on the stage and molecular characterization. The prognosis for patients with breast cancer BM is poor with a median OS ranging from 5.5 to 14.1 months and survival rates decrease from 60% at 1 year to 17% at 5 years following a BM diagnosis.^51,52,54^

Breast cancer is classified based on hormone receptor (HR) positivity and human epidermal growth factor receptor 2 (HER2) status. Tumors may be positive for estrogen receptor (ER) and progesterone receptor, HER2, or they may be triple-negative (TN) (lacking ER, progesterone receptor, and HER2). *BRCA1* mutations are commonly associated with TN tumors and high rates of BM (up to 44%), while *BRCA2* mutations often result in HR-positive/HER2-negative tumors with lower BM rates (16.7%).^55^

Among breast cancer patients, the most common subtype is luminal A (HR+/HER2−) (72.4%), followed by TN (11.8%), luminal B (HR+/HER2+) (10.9%), and Her2 enriched (HR-/HER2+) (4.8%).^56^ Patients with HER2+ and TN tumors have a higher incidence of BM, particularly at initial diagnosis and during their lifetime (28.0% and 30.8%, respectively).^56,57^ In patients with metastatic disease to any site, the incidence of BM at initial breast cancer diagnosis is 11.37% in HR−/HER2+ and 11.45% in TN.^58^ Along with the incidence of BM at initial diagnosis, HER2+ and TN molecular subtypes also have a significant risk of developing BM in their lifetime with respective rates of 50% and 25%–46%.^15,52^ Patients who have metastatic TN breast cancer have a high incidence of early BM (17% at 1 year and 25% at 2 years) and a median interval from extracranial metastasis to BM of 10 months.^59^ Interestingly, HER2+ tumors were previously thought to be a poor prognostic factor, but have been associated with longer survival rates in breast cancer BM patients as newer HER2-targeted therapies improve clinical outcomes due to improved CNS penetration.^53^ HR positivity is associated with longer BM-free survival compared to other subtypes (HR+ 75 months; HER2 + 39 months; TN 30 months).^22^ Other molecular predictors, such as the presence of Ki-67 levels over 30%, can also impact BM risk and OS.^60^

Current NCCN guidelines only recommend screening for BM in breast cancer patients with suspicious neurological symptoms in advanced-stage (IV) or recurrent disease.^9^ Predictors of BM in breast cancer patients include: Younger age, presence of visceral metastases, number of metastasis sites, and HER2+ and TN molecular subtypes.^12,61^ However, the rates of BM in these subgroups are not high enough to justify screening according to the current consensus.^62^ Nonetheless, these data are generated from smaller studies with conflicting recommendations.

Two clinical trials, NCT04030507 and NCT03881605 are evaluating the role of MRI screening in patients with advanced, metastatic, or inflammatory breast cancer. The findings from these studies may lead to revised screening recommendations based on molecular subtype.

Unfortunately, BMs in breast cancer patients are often discovered late, when they are symptomatic and clinically advanced, leading to poor prognosis and aggressive management.^63–65^

### Melanoma

Approximately 40%–50% of melanoma patients will develop BM.^15,16,66,67^ The time from primary diagnosis to BM ranges from 30 to 32 months and the cumulative risk of BM at 5 years following diagnosis is 7%.^52,68,69^ Interestingly, the first site of relapse is the brain for up to 20% of melanoma patients.^70^ Historically, melanoma BM has been associated with poor prognoses and OS of approximately 4 months.^69,71^ However, the advent of immunotherapy and BRAF/MEK inhibitors has improved OS in recent years extending survival up to 13 or 23 months.^72^ Early detection and treatment of melanoma BM remains crucial for improving outcomes.

Melanoma progression and the development of BM are influenced by microenvironmental cues and molecular factors, including mutations in *BRAF*, *NRAS*, and *PTEN*, and activation of the PI3K/AKT (by chemokine ligand CCR4) and JAK/STAT3 pathways.^68^ CCR4 activation, for instance, triggers PI3K/AKT activation and contributes to the adhesion of primary tumor cells to the brain.^73^

*BRAF* mutations and RAS/RAF/MEK pathway activation are key drivers of tumor growth and metastasis, especially when paired with *PTEN* loss.^74,75^ Up to 37% of patients with melanoma carry *BRAF* mutations irrespective of BM status.^76^ Importantly, patients with *BRAF* mutations are at a higher risk of BM incidence and approximately 29%–56% of melanoma patients with BM have *BRAF* mutations in their primary tumors.^52,77,78^ In particular, melanomas with *BRAF*-V600 mutations have an increased risk of BM and it is estimated that *BRAF*-V600 is found in approximately 14% of melanoma BM.^78^ Importantly, patients with *BRAF* mutations have longer median survival than those without, likely due to targeted therapies against *BRAF*-mutant melanoma with excellent CNS penetration and immune checkpoint inhibitors.^79,80^

Other genes and proteins, such as *KRAS*, *NGFR* (nerve growth factor receptor), and *NRAS* (neuroblastoma ras viral oncogene homolog), are also implicated in melanoma BM development. *C-KIT* mutations are present in 11% of melanoma patients with BM, but do not significantly affect survival. Interestingly, the genetic similarity, found between melanoma BM and extracranial metastases suggests these *KRAS* mutations may be found in the primary melanoma site and can indicate the necessity for BM surveillance.^81^*NRAS* mutations causing constitutive expression of the NRAS protein leading to unregulated cell growth and division can be found in 18%–22% of melanoma BM patients and have been linked to an increased cumulative incidence of BM.^52,78^ Furthermore, the expression of proteins such as NGFR mediates stages of tumor progression in melanoma BM and high levels correlate with the development of BM.^82,83^

NCCN guidelines recommend initial screening for BM in metastatic or advanced-stage melanoma, non-metastatic melanoma patients who develop neurological symptoms, and/or when the presence of BM may inform the treatment course.^10^ Follow-up MRI is recommended every 2–3 months to monitor treatment response and inform future management decisions, such as stereotactic radiosurgery (SRS) or resection.^84^ Although early-stage melanoma does not currently have screening recommendations, evidence suggests that FDG-PET-CT scans based on gene stratification may optimize BM detection.^85^

The guidelines for follow-up BM screening after a negative MRI are unclear, with limited literature available. The NCCN guidelines suggest periodic MRI for high-risk metastatic melanoma stage IIIC and above. Recent data suggests that approximately one-third of BM diagnoses are asymptomatic and diagnosed on follow-up MRI within 2 years of melanoma diagnosis. The discovery of asymptomatic BM may change management in affected patients. Furthermore, interval scans may prompt treatment changes in up to 89% of cases supporting the use of MRI for surveillance.^86^

## Discussion

Screening for BM is crucial for patients with lung cancer, breast cancer, or melanoma. Physicians must consider risk factors and molecular characteristics when assessing patients for BM. The molecular subtypes with the highest BM incidence are *EGFR*-mutated NSCLC and BRCA1, TN, and HER2+ breast cancers. Similarly, BM is more likely to present asymptomatically at diagnosis in oncogene-addicted NSCLC and *BRAF*-mutated melanoma, which emphasizes the importance of molecular subtype-based screening. Conversely, while most common, luminal A breast cancer has the lowest incidence indicating a possible reduced need for early screening in about 70% of breast cancer patients. European screening standards recommend more frequent screening for oncogene-addicted NSCLC patients, and clinical trials are investigating screening for BM in HR+, HER2+, and TN breast cancers.^22,87,88^

The current NCCN screening guidelines recommend brain imaging for NSCLC, SCLC, and metastatic or advanced-stage melanoma, but not for any breast cancer subtype (Table 3).^8–11^ While there are ongoing clinical studies investigating the role of BM screening in HR+, HER2+, TN, and inflammatory breast cancers, to date data are conflicting and require further investigation.^44,45^ Due to a relative paucity of data, further clinical trials incorporating molecular data into screening guidelines for BM are necessary to rigorously evaluate the benefits of molecular-based BM screening recommendations. The absence of guidelines for follow-up imaging for asymptomatic breast cancer and NSCLC patients after an initial negative MRI highlights the need for more research in this area, especially as many patients who are initially negative for BM go on to develop BM later in the disease course.^48^ Additionally, adjusting guidelines to encompass formal categorization of high-risk molecular groups may reduce missed BM due to premature screening in groups with a later time to development of BM.^89,90^

**Table 3.** National Comprehensive Cancer Network Screening and Surveillance Recommendations

Cancer Type	Screening Recommendations	Surveillance Recommendations
Non-small cell lung cancer	Initial screening brain MRI with contrast for stages II, III, and IV Stage IB optional	Not indicated after completion of definitive therapy unless symptomatic or there is evidence of recurrence
Small cell lung cancer	Recommended at initial diagnosis for all stages of SCLC or combined NSCLC/SCLC	Brain MRI repeated after completion of primary treatment and continued surveillance every 3–4 months until 1 year after treatment, then every 6 months until 2 years after treatment, and then imaging if symptomatic or evidence of recurrence
Breast cancer	Not recommended unless recurrent or stage IV and suspicious CNS symptoms are present	Not indicated
Melanoma (cutaneous)	Screening with brain MRI recommended for stage IV or recurrence with distant metastases Optional for stage IIIB/C/D	Imaging in the setting of suspicious symptoms. Consider imaging every 3–12 months for 2 years and then every 6–12 months for another 3 years (stage IIB–IV) or more frequently for patients with prior brain metastases

Early detection and control of BM can mitigate the increased healthcare costs, economic burden, and decreased quality of life (QoL) that come with a BM diagnosis.^91^ Monthly healthcare costs have been shown to increase 4-fold from $5983 to $22 645 after BM diagnosis in NSCLC patients.^92^ Furthermore, healthcare utilization also increased, with a 3-fold increase in outpatient visits and 6-fold increase in hospitalizations.^92^ The increase in healthcare costs following the diagnosis of BM is mainly driven by pharmaceutical expenditures, followed by inpatient visits, and then outpatient visits.^92^ Healthcare costs are highest in melanoma patients, followed by breast and then lung cancer patients.^93^ The cost of treating symptomatic BM is higher than for asymptomatic BM and routine screening may increase cost savings in the long term and improve treatment outcomes. Indeed, one retrospective study of patients who underwent SRS for BM management demonstrated that performing routine MRI surveillance after SRS saved an estimated $1326 per patient.^94^ This study also found that the cost of treating symptomatic BM was $41 700, compared to $29 743 for asymptomatic BM, and the predominant contributor to higher costs in symptomatic patients was neurosurgical intervention.^94^

There is limited research on early screening for the detection of BM and how it would impact morbidity, mortality, and treatment plans. Although there are few clinical trials investigating the role of early MRI screening, some studies suggest there is a benefit to identifying BM in asymptomatic patients as they are likely to have fewer and smaller BM.^89,95^ Maurer et al. show that among HER2+ breast cancer patients detected to have BM, the 40% who presented asymptomatically demonstrated better survival (HR 0.49, 95% CI 0.25 to 0.94).^96^ Similarly, Laakmann et al. show longer median OS in asymptomatic HER2+ breast cancer patients (10.4 vs. 6.9 months).^95^ Further progression and growth of BM limit local treatment options for patients as SRS procedures may not be appropriate for extensive CNS involvement; candidates have few and small metastases <3 cm in size.^96^ Patients undergoing SRS have improved survival with BM measuring <10 mm in diameter and a 100% rate of local control when measuring <6 mm.^97^ Additionally, further work is required to clarify the penetrance of systemic therapies, with some studies suggesting high-intracranial efficacy with next-generation tyrosine kinase inhibitors and immune checkpoint inhibitors in asymptomatic patients.^98^ For instance, melanoma patients with symptomatic BM are relatively resistant to systemic therapy with the combination of nivolumab-ipilimumab, while this combination provides a significant clinical benefit to those who are asymptomatic.^99^

Patients at high risk for BM can benefit from targeted surveillance imaging to reduce false positives and associated costs. These risks may be affected by higher age, number of lesions, and extracranial spread.^26^ Molecular characteristics can help define high-risk groups, but demographics and racial differences also play a role in BM incidence, morbidity, and mortality. Middle-aged women are more likely to develop BM, with higher incidence and mortality rates in lung cancer, while men have higher incidence of melanoma.^4,100^ African Americans have a higher population-based incidence of BM for lung cancer, breast cancer, and melanoma compared to White patients and are more likely to experience de novo BM or cancer that has already metastasized upon primary diagnosis.^17,101^ Additionally, disproportionately high morbidity and in-hospital mortality are seen in African American women with BM.^100^

To revise BM screening protocols, physicians should assess their patients’ risk holistically to balance molecular predisposition, sex, race-based trends, and prognostic factors. The efficacy of known treatments within molecular subtypes may also be a factor considered in the revision of screening protocols. For instance, early identification of *BRAF*-mutated primary melanoma indicates the use of first-line immunotherapy with immune checkpoint inhibitors (anti‐CTLA4/anti‐PD1/combination) which has been shown to more significantly reduce BM incidence, TPDBM, and OS when compared to those treated with targeted therapies (inhibitors of BRAF/MEK/combination)^102^ therefore, potentially delaying or reducing the need for early BM screening in this group.^76^ Conversely, mutations in melanoma such as *NRAS* Q61K/L, *MEK1* P124, *RAC1* P29S, or *BRAF* L514K are intrinsically resistant to treatment and are associated with progression to BM, therefore, necessitating more screening.^82^ Furthermore, early identification of BM is becoming more relevant with the increasing number of targeted therapies and immunotherapies with good efficacy for BM. Recent studies have demonstrated that targeted therapy with inhibitors of the cyclin-dependent kinase pathway, such as palbociclib, demonstrates intracranial benefit for patients with cyclin-dependent BMs.^103^ Immune checkpoint inhibitors also shows promise in the treatment of BM. A recent study demonstrated that the use of the immune checkpoint inhibitors, combined nivolumab, and ipilimumab, had intracranial clinical benefit for 57% of patients with metastatic melanoma.^104^ While pembrolizumab demonstrates activity in NSCLC BM with increased PD-L1 expression.^105^ In addition to research on the treatment of BM, there has also been recent interest in the prevention of BM, with a recent clinical trial demonstrating that temozolomide, in addition to T-DM1 for HER2+ breast cancer, has the potential for BM prevention.^106^

While the present paper focuses on how the molecular characterization of primary tumors should guide screening practices prior to BM diagnosis, there is interest in the characterization of circulating tumor cells in the peripheral blood through next-generation sequencing. The identification of molecular signatures which confer “metastatic competency” and markers prognostic for BM has the potential to impact treatment after BM development.^107,108^ There has been much advancement in technology used to identify and characterize circulating tumor cells and as knowledge surrounding genetic drift and targetable molecules becomes more available, it will be applied in clinical decision-making.^109^ The current paper posits a relationship between primary tumor characteristics and propensity for spread to the CNS, but it is important to note heterogeneity may exist between primary or extracranial and intracranial metastases and can potentially alter therapeutic interventions. For instance, Brastianos et al. identify 46 of 86 (53%) patients with alterations in BM with associated sensitivity to PI3K/AKT/ mTOR, cyclin-dependent kinase, and HER2/EGFR inhibitors not detected in the primary tumor.^110^ While the molecular subtype of a primary tumor may indicate need for more intensive screening, further investigation is warranted into the effects of somatic variants on CNS tropism and how treatments can be tailored to the unique molecular profile of BM.

## Conclusion

Primary lung cancer, breast cancer, and melanoma are the top 3 causes of BM, and the consequence of CNS progression is an increase in patient morbidity and mortality. Current screening practices for brain metastasis are conservative and often limited to patients with advanced-stage cancers. As our capabilities to characterize tumors continue to improve, the identification of molecular subgroups can help identify patients who are at a higher risk for brain metastasis and help reform screening recommendations.
